# The Role of Infected Cell Proliferation in the Clearance of Acute HBV Infection in Humans

**DOI:** 10.3390/v9110350

**Published:** 2017-11-18

**Authors:** Ashish Goyal, Ruy M. Ribeiro, Alan S. Perelson

**Affiliations:** 1 Theoretical Biology and Biophysics, Los Alamos National Laboratory, Los Alamos, NM 87545, USA; ruy@lanl.gov (R.M.R.); asp@lanl.gov (A.S.P.); 2 Laboratório de Biomatemática, Faculdade de Medicina da Universidade de Lisboa, 1649-028 Lisboa, Portugal

**Keywords:** hepatitis B virus, hepatocyte turnover, cytokine-mediated cure of infected cells, cellular proliferation

## Abstract

Around 90–95% of hepatitis B virus (HBV) infected adults do not progress to the chronic phase and, instead, recover naturally. The strengths of the cytolytic and non-cytolytic immune responses are key players that decide the fate of acute HBV infection. In addition, it has been hypothesized that proliferation of infected cells resulting in uninfected progeny and/or cytokine-mediated degradation of covalently closed circular DNA (cccDNA) leading to the cure of infected cells are two major mechanisms assisting the adaptive immune response in the clearance of acute HBV infection in humans. We employed fitting of mathematical models to human acute infection data together with physiological constraints to investigate the role of these hypothesized mechanisms in the clearance of infection. Results suggest that cellular proliferation of infected cells resulting in two uninfected cells is required to minimize the destruction of the liver during the clearance of acute HBV infection. In contrast, we find that a cytokine-mediated cure of infected cells alone is insufficient to clear acute HBV infection. In conclusion, our modeling indicates that HBV clearance without lethal loss of liver mass is associated with the production of two uninfected cells upon proliferation of an infected cell.

## 1. Introduction

Hepatitis B virus (HBV) is a highly prevalent infection with approximately 350 million chronic carriers world-wide, causing about 1 million deaths each year [1]. Despite the existence of a safe and efficacious vaccine, the current high prevalence, high mortality and lack of effective antiviral therapy make HBV a severe health problem. Much effort has been expended in the past three decades to comprehensively understand HBV [2,3,4,5,6,7,8,9,10,11,12,13,14,15,16,17]. In this quest, in vitro experiments along with in vivo experiments in ducks, woodchucks, mice and chimpanzees have significantly improved our understanding of HBV infection and its interaction with the immune system. Though experiments in animal models and in vitro have provided important insights, some characteristics of HBV infection are different in humans. One of the major differences is the higher number of covalently closed circular DNA (cccDNA) molecules per infected cell in animal models compared to humans [18,19,20,21,22].

In particular, our understanding of acute HBV infection in humans is still very limited due to the lack of data, as it is extremely difficult to find and sample people at this stage of infection. Nonetheless, some data is available [6] and mathematical models can be used along with this data to investigate possible mechanisms underlying the clearance of acute HBV infection.

In the past, mathematical models have been used extensively to study the viral dynamics of infections such as HBV, hepatitis C virus (HCV) and human immunodeficiency virus (HIV) [5,6,13,23,24,25,26,27,28,29,30,31,32,33,34,35,36,37,38]. Here, we propose new mathematical models and investigate different mechanisms related to the natural clearance of acute HBV infection, using a longitudinal dataset of six acutely HBV infected individuals, who naturally cleared the infection [6].

Although the majority of adults infected with HBV clear infection [39,40], how this occurs is not fully understood. The clearance of acute HBV infection correlates with the strengths of the cytolytic and non-cytolytic immune responses [41,42]. It is thought that cytotoxic T lymphocytes (CTL or T-cells) clear infection by inducing the death of infected cells while non-cytolytic mechanisms involve antiviral cytokines, such as interferon-γ (IFN-γ) and tumor necrosis factor-α (TNF-α), that inhibit intracellular HBV replication and thus assist in the control of HBV infection [9,43,44,45,46,47]. There are two other mechanisms whose importance in the clearance of acute HBV infection has been debated [9,44,48,49]. The first involves the substantial loss of cccDNA during proliferation of infected cells [50,51,52,53], while the second assumes that cytokines can also induce the degradation of cccDNA (in addition to the inhibition of the intracellular HBV replication), which then leads to the cure of infected cells (this mechanism is referred to as cytokine-mediated cure of infected cells) [44]. The loss of cccDNA upon cell division could occur if cccDNA failed to be reincorporated into the nucleus when the nuclear membrane reforms during mitosis. Also, cccDNA can be lost by dilution during cell proliferation and if the cccDNA copy number were small, due to random segregation of cccDNA between daughter cells some cells may fail to inherit any cccDNA. It is important to understand the role of these additional mechanisms in the resolution of acute HBV infection. This is because acute HBV infection can be cleared in 8 to 12 weeks in the presence of limited immune responses [54,55] despite nearly all hepatocytes being infected with HBV at the peak of acute infection [4,22,55,56], with each infected cell containing at least one cccDNA molecule with an estimated half-life of around 50 days [57]. In vivo and in vitro experiments have hinted that these two additional mechanisms could be important in the clearance of HBV acute infection [44,51,52,58,59]; however, these studies were not conclusive [60,61]. In addition, a recently conducted computational study showed the importance of cccDNA loss during cellular proliferation in the process of non-destructive clearance of HBV infection [24]. To investigate this with human data, we developed models to test three hypotheses on the effect of infected cell proliferation: it produces (i) two uninfected daughter cells, (ii) two infected daughter cells or (iii) one uninfected and one infected daughter cell. We also tested if cytokine-mediated cure of infected cells is able to clear acute HBV infection.

## 2. Materials and Methods

### 2.1. Patient Data

Whalley et al. [6] identified 7 patients infected in the acute phase of HBV infection. Data from patient 7, treated with the reverse transcriptase inhibitor lamivudine, is not representative of the natural course of the disease and was excluded from the present study. HBV DNA in serum was measured longitudinally in the remaining six patients for up to 150 days [6]. Ultimately, all these six patients cleared HBV infection. We assume that the effect of immune-mediated clearance starts at the peak of viremia for each patient, as we did before [25]. The patient data is provided in the supplementary information (Table S1).

### 2.2. Mathematical Models

The liver consists mostly of hepatocytes and a variety of nonparenchymal cells. Hepatocytes are responsible for many of the crucial functions of the liver and are the primary target for HBV. The liver, if damaged, can regenerate and thus hepatocytes, even though highly differentiated, retain the capacity to proliferate. One criterion that we use to access the realism of a model is whether the proliferative response to infection maintains the number of hepatocytes above a critical threshold needed for adequate functioning. Thus, it is important to know the number of hepatocytes in a healthy liver. The literature suggests that hepatocytes are ~60% of the 2 × 10^11^ cells in an adult liver [62,63,64,65].

#### 2.2.1. Models Characterizing Different Outcomes of the Cellular Proliferation of Infected Cells

We develop mathematical models to investigate three scenarios regarding infected hepatocyte proliferation. When an infected cell divides it can generate (i) two uninfected daughter cells (Model 1), (ii) two infected daughter cells (Model 2), and (iii) one uninfected and one infected daughter cell (Model 3).

T, I and V represent concentration of target cells, infected cells and the concentration in serum of HBV DNA, respectively. Infection occurs with infectivity rate constant k while infected cells die at per capita rate δ. The per infected cell production rate of HBV is denoted by $p_{V}$ and $c_{V}$ is the clearance rate of HBV. Here, θ is the fraction of liver cells that cannot be infected (i.e., nonparenchymal cells) in the total liver cell population and H is the total liver cell population before infection, which includes both hepatocytes and nonparenchymal cells. Both θ and H are assumed to be constants. Additionally, we assume that uninfected and infected hepatocytes proliferate according to a logistic growth law with $r_{T}$ and $r_{I}$, respectively. By using a logistic growth law, the total number of liver cells can never grow larger than H. We simplify our model by assuming $r=r_{T}=r_{I}$ to reduce the number of unknown parameters. With these assumptions, the three models are described by the following equations

*Model 1 (M1)*-Proliferation of an Infected Cell Produces Two Uninfected Cells

$$\frac{dT}{dt}=\overset{\begin{array}{c} Each\;uninfected\;cell\;that\;undergoes\\ proliferation\;produces\;two\\ uninfected\;cells\;or\;one\;additonal\;uninfected\\ cell\;on\;top\;of\;the\;one\;that\;underwent\;proliferation \end{array}}{\overset{︷}{rT\left(1-\frac{T+I+\theta H}{H}\right)}}+\overset{\begin{array}{c} Each\;infected\;cell\;that\;undergoes\\ proliferation\;produces\;two\\ uninfected\;cells \end{array}}{\overset{︷}{2rI\left(1-\frac{T+I+\theta H}{H}\right)}}$$

(1) $$\frac{dI}{dt}=\overset{\begin{array}{c} Infection\;of\\ target\;cells \end{array}}{\overset{︷}{kVT}}-\overset{\begin{array}{c} Clearance\;of\\ infected\;cells \end{array}}{\overset{︷}{\delta I}}-\overset{\begin{array}{c} Loss\;of\;infected\;cells\\ undergoing\;proliferation \end{array}}{\overset{︷}{rI\left(1-\frac{T+I+\theta H}{H}\right)}}$$

$$\frac{dV}{dt}=\overset{\begin{array}{c} Production\;of\\ virus\;from\\ infected\;cells \end{array}}{\overset{︷}{p_{V}I}}-\overset{Clearance\;of\;virus}{\overset{︷}{c_{V}V}}$$

*Model 2 (M2)*-Proliferation of an Infected Cell Produces Two Infected Cells

$$\frac{dT}{dt}=\overset{\begin{array}{c} Each\;uninfected\;cell\;undergoing\\ proliferation\;producing\;one\\ additional\;uninfected\;cell \end{array}}{\overset{︷}{rT\left(1-\frac{T+I+\theta H}{H}\right)}}-kVT$$

(2) $$\frac{dI}{dt}=\overset{\begin{array}{c} Each\;infected\;cell\;undergoing\\ proliferation\;producing\;one\\ additional\;infected\;cell \end{array}}{\overset{︷}{rI\left(1-\frac{T+I+\theta H}{H}\right)}}+kVT-\delta I$$

$$\frac{dV}{dt}=p_{V}I-c_{V}V$$

*Model 3 (M3)*-Proliferation of an Infected Cell Produces One Uninfected and One Infected Cell

$$\frac{dT}{dt}=\overset{\begin{array}{c} Each\;hepatocyte\;undergoing\\ proliferation\;producing\;one\\ additional\;uninfected\;cell \end{array}}{\overset{︷}{r\left(T+I\right)\left(1-\frac{T+I+\theta H}{H}\right)}}-kVT$$

(3) $$\frac{dI}{dt}=kVT-\delta I$$

$$\frac{dV}{dt}=p_{V}I-c_{V}V$$

*M3* can also represent a model where cellular proliferation results, on average, in the loss of cccDNA 50% of the time and preservation of cccDNA 50% of the time.

#### 2.2.2. Model Incorporating Cytokine-Mediated Cure of Infected Cells

We also investigate two additional models in which there is cytokine-mediated cure of infected cells.

*Model 4 (M4)*-Proliferation of an Infected Cell Produces Two Infected Cells and There Is Cytokine-Mediated Cure of Infected Cells

In this model, we assume that proliferation of an infected cell produces two infected cells and that there is cytokine-mediated degradation of cccDNA, which results in the loss of infected cells at rate ρI, without cell death. This model, *M4*, is derived by subtracting the term ρI in the second equation and adding it in the first equation of *M2*.

*Model 5 (M5)*-Proliferation of an Infected Cell Produces Two Uninfected Cells and There Is Cytokine-Mediated Cure of Infected Cells

Finally, in this model we assume that proliferation of an infected cell produces two uninfected cells and there is cytokine-mediated cure of infected cells (the term ρI). Thus, we subtract and add the term ρI in the second and first equation respectively of *M1*, and refer to it as *M5*.

### 2.3. Parameter Values and Simulation Procedure

We take $H=13.6\times 10^{6}\;\mathrm{cells}/\mathrm{mL}$ as in prior studies [66]. In chimpanzees, mice and ducks, 95–99% of hepatocytes are infected at the peak of acute infection [19,26]. In addition, from a modeling study the mean fraction of HBV infected hepatocytes in humans at the peak of infection has been estimated to be at least 95% [66]. As the human viral load data from [6] that we analyze was first collected near peak viremia, we let t=0 refer to the time of peak viremia and the corresponding viral load at peak as V(0). At this time, we assume I(0)=0.95(1−θ)H and T(0)=0.05(1−θ)H, where I(0) and T(0) are the infected and uninfected hepatocyte populations respectively at time t=0, i.e., the peak of viral load in acute infection. In addition, (1−θ) is the fraction of the total number of cells in the liver (H) that are hepatocytes, and thus HBV targets. We first simulated models *M*1–*M*3 and estimated the default value of the virus infectivity parameter as the maximum value of k under which all patients satisfy all the model selection criteria (discussed in the Section 2.4) under any one of the three models. The default value of k was estimated to be $0.55\times 10^{-10}\;\mathrm{mL}/\mathrm{copies}\cdot \mathrm{day}$ (see Tables S2–S5). In Section 3.5.1, we perform sensitivity analysis on the choice of virus infectivity, k, and the size of the initial infected and uninfected hepatocyte populations, I(0) and T(0). Furthermore, we assumed θ=0.4 corresponding to 60% of liver cells being hepatocytes [62,63,67].

In order to estimate the four unknown parameters, namely, r, δ, $p_{V}$ and $c_{V}$, we used the Levenberg–Marquardt algorithm embedded in the optimization procedure “lsqnonlin” in MATLAB R2016b (The Mathworks, Inc., Natick, MA, USA) to fit the different models to the viral load data obtained from the 6 patients. We also estimate the parameter ρ associated with models *M4* and *M5* and constrain ρ to be between 0.001 and 0.35/day [24]. To avoid local minima, we perform fitting with 100 random initial parameter guesses for each patient, and then choose the parameters with the lowest error, where error is given by $RSS=\sum_{i=1}^{n_{V}}\left(\log10(V_{i}\right)-\log10({\bar{V}}_{i}){)}^{2}$, and $i=1,\;2\ldots ,n_{V}$ refer to the viral load data points, $\bar{V_{i}}$, and the analogous value given by our model is $V_{i}$ [68]. Note that in comparing the initial guesses, we are comparing models with the same number of parameters on the same data set.

In the estimation procedure, we constrained the parameter search over biologically reasonable ranges. Thus, we assumed a minimum value of δ=0.001/day [69,70] but left the maximum value unconstrained. In addition, the value of r was constrained to be between 0.001 and 3.4/day [71,72]. Similarly, the value of $c_{V}$ was constrained to be between 0.67 and 4.2/day [13,66]. Cytokines are recruited during the clearance of acute infection and they are present post-peak in acute HBV infection contributing to the inhibition of HBV replication [54]. Therefore, we assume that the maximum value of viral production, $p_{V}$ occurs at the peak of the infection. This maximum value is determined by the fact that dV/dt =0 at peak viremia and therefore the production rate of HBV at t=0, i.e., at peak viremia, is $c_{V}V\left(0\right)/I\left(0\right)$. We estimate $p_{V}$ from the data fitting and since that value is less than the maximum value at *t = 0*, we take this to mean that non-cytolytic inhibition of viral production by cytokines may be occurring. The average efficacy of the inhibition of HBV replication by cytokines can be given by $1-p_{V}I\left(0\right)/c_{V}V\left(0\right)$.

To compare models, we use the corrected Akaike information criterion (AIC_C_) calculated as AIC_C_ = (nln(RSS/n)) + (2mn/(n − m − 1)), where *m* is the number of unknown parameters and *n* is the number of data points used in the fits [68,73,74]. We also calculate the total AIC_C_ with total residual sum of squares over all patients as RSS, while *n* and *m* as the total number of data points and unknown parameters over all patients, respectively. The smaller the AIC_C_, the better the model is supported by the data. However, when the AIC_C_ difference between two models is less than 2, both models have equal support. When this difference is between 4 and 7, then the evidence for better support of one model over another is weak. If it is more than 10, then the evidence is strong [75,76].

### 2.4. Criteria to Determine the Biological Plausibility of the Model

Apart from a good-fit to the viremia data, the desired outcomes include clearance of the infection while maintaining liver integrity and complete elimination of the infected cell population to avoid relapse. The first criterion of non-destructive clearance is the amount of hepatocyte turnover (HT), defined as the number of hepatocytes (normalized to the total number of hepatocytes in an uninfected adult liver) lost during the process of clearance. HT is measured as $\;\int_{0}^{\infty }\frac{\delta I\left(t\right)}{\left(1-\theta \right)H}dt$, which has been estimated to be in the range of 0.35–3 [14,24,25,53,77] during immune-mediated clearance in 12 weeks. Alongside, HT should be as low as possible because persistent apoptosis over a long period also serves as a mechanism of liver injury and carcinogenesis [78]. Therefore, we use HT < 3 as a measure of non-destructive clearance of acute HBV infection in humans, with a preference for HT to be as low as possible to avoid the possibility of liver injury and/or scarring. The second criterion requires that the infected cell population decrease to less than one cell to reflect true clearance of HBV infection. We also calculate the number of hepatocytes (normalized to the total number of hepatocytes in an uninfected adult liver) cured by cytokines in the process of cytokine-mediated cure of infected cells, i.e., non-destructive turnover as NDT = $\int_{0}^{\infty }\frac{\rho I\left(t\right)}{\left(1-\theta \right)H}dt$.

In studies of liver transplant from living donors, a hepatic resection leaving more than 20–30% of the original liver volume is considered safe and allows liver regeneration in the donor [79,80,81]. Kishi et al. studying more than 300 patients with extended right lobe hepatectomies found that having a liver remnant volume >20% is sufficient for safe hepatic resection [81]. As there can also be further hepatocyte cell death after liver resection due to ischemia reperfusion injury [79], which does not occur during HBV infection, we assumed that in acute HBV infection having 20% of the original hepatocyte number (i.e., 0.12*H*) is sufficient for maintaining the essential functions of the liver, but anything less than 20% can be fatal. Thus, a second model criterion is having the hepatocyte number remain above 0.12*H*. We note that in terms of liver cell population size, this critical level corresponds to at least 52% of the liver cells still remaining (the sum of (i) θH=0.4H from the constant nonparenchymal cell population, and (ii) 0.12H from the hepatocyte cell population). Based on this we will reject models in which the predicted liver cell number falls below 52% (or the hepatocyte number falls below 20%) of their original numbers.

## 3. Results

### 3.1. Fitting to Viral Load Data Alone Does Not Differentiate Models

**Figure 1:**
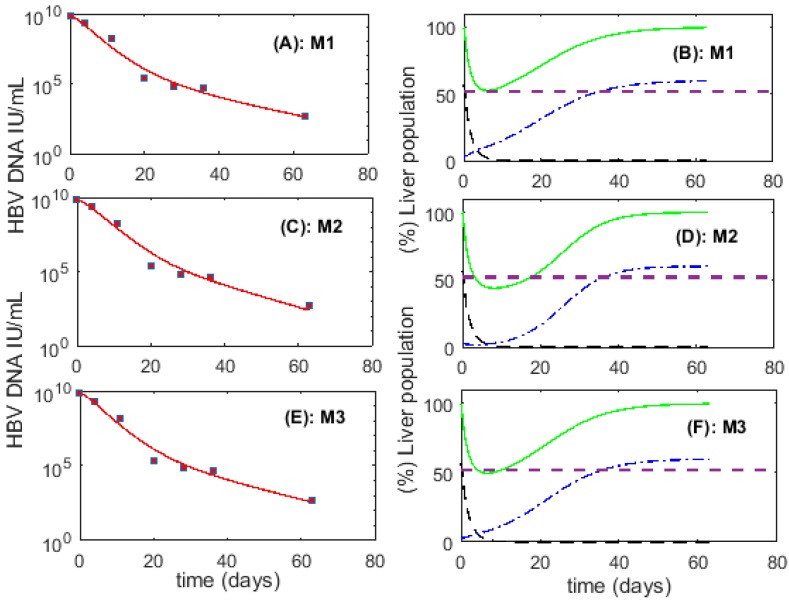
Panels (**A**), (**C**) and (**E**) represent the best fit (line) to hepatitis B virus (HBV) viremia data (squares) for patient 1 using *M1*, *M2* and *M3* respectively; panels (**B**), (**D**) and (**F**) represent (%) infected cell population (*I*, black dashed line), (%) uninfected cell population (*T*, blue dashed dot line), and (%) total liver cell population (100(I+T+θH)/H, green solid line) corresponding to the best fit of HBV viremia data. The dotted horizontal line represents the critical threshold for the liver cell population during the process of infection clearance (0.52*H*). Best-fit parameter values are given in Table S7.

First, we checked whether the fit of *M1*, *M2* and *M3* to the HBV viremia data is sufficient to differentiate between the fates of cccDNA during cell division of infected cells by comparing the AIC_C_ for models 1, 2 and 3 (Table S6). With lower AIC_C_ for each patient (and also lower total AIC_C_), our simulations showed better support for *M1* over *M2* and *M3* for all 6 patients. However, we also found that the differences in AIC_C_ among the models for the 6 patients is too small to reach definite conclusions (shown for P1 in Figure 1A,C,E). Therefore, we cannot choose the best model just based on fitting to patient HBV viremia data.

The experimental data show that all six patients cleared acute HBV infection without critical liver destruction and therefore in the next section we track the infected cell population to check for the true clearance of acute HBV infection in these 6 patients.

### 3.2. Biological Constraints Indicate that Proliferation of Infected Cells is More Likely to Produce Two Uninfected Cells

We next considered the predictions of the three models concerning hepatocyte turnover and the size of the liver throughout acute infection. We impose the conditions that the hepatocyte turnover should be less than 3 and that the liver cell population during the clearance (I+T+θH) never becomes smaller than 52% of the total liver cell population before infection (H).

The best-fits of the models to the data revealed that *M1*, where an infected cell produces two uninfected cells is the only model which not only produces a good fit to the HBV viremia (see total AIC_C_ in Table S6) but also maintains a functioning liver for all patients during the process of infection clearance (Table 1). In particular, patient 1 (P1) plays an important role in distinguishing the three models because we observed more than 5 log (first-phase) decrease in HBV viremia in this patient. In contrast to *M1*, models *M2* and *M3* showed significant liver destruction/dysfunction during the clearance of acute HBV infection, in particular for P1 (see Figure 1B,D,F and Table 1). Model 2, where proliferation of an infected cell generates two infected cells, results in a non-functioning liver (with the total liver cell population during clearance (I+T+θH) decreasing below 52% of the total liver cell number before infection (H)) for all patients (see bold and underlined numbers in Table 1). Over all patients, *M1* also resulted in the clearance of acute HBV infection with lowest mean HT (0.87) compared to *M2* (1.14) and *M3* (1.05).

### 3.3. Cytokine-Mediated Cure of Infected Cells is Insufficient to Achieve Non-Destructive Clearance of Acute HBV Infection

Another process hypothesized to assist clearance of HBV infection is cytokines mediated destabilization of cccDNA (and possibly cure of infected cells) [44,49,58]. Therefore, we investigated this mechanism under model *M4* (via the term ρI in the model). In this model, we also assumed that infected cell proliferation results in two infected cells, because we want to test the ability of cytokine-mediated cure to clear HBV infection by itself (see Section 3.4 for another possibility).

We find that the data does not support this model as well as *M1*–*M3*, since the total AICc for *M4* (81.9) is more than 30 points higher than the AICc score for models *M1*–*M3* (Table S6). Moreover, we find that cytokines-mediated cure of HBV infected cells is insufficient to clear acute HBV infection in all six patients without liver dysfunction (see P1 in Table 2). We also find that the number of infected cells that become uninfected due to cytokine-mediated cccDNA degradation is very small (and close to zero) for patient 1 (non-destructive turnover (NDT) in Table 2 and ρ in Table S8). This could be due to the observed >5 log first-phase decrease in HBV viremia in patient 1.

### 3.4. Cytokine-Mediated Cure of Infected Cells and cccDNA Loss during Cellular Proliferation

Above, we tested whether cccDNA loss during cellular proliferation is more important than cytokine-mediated cure of infected cells in clearing acute HBV infection. We found that cccDNA loss during cellular proliferation had a more critical effect; however, it is also possible that these two mechanisms act in concert. We investigated this phenomenon with model *M5*. The results are summarized in Table 2.

The results for *M5* (which is the same as *M1* but incorporating cytokine-mediated cure of infected cells) are similar to those of model *M1*. For example, both models reproduced the viral dynamics without destruction of the liver (Table 1 and Table 2).

However, the total AIC_C_ for *M1* (45.8) was much smaller than for *M5* (77.7), which has one additional parameter per patient, indicating lack of support for the more complex model. Therefore, *M1* that did not include cytokine-mediated cure of infected cells performed better than the model incorporating cytokine-mediated cure of infected cells. In summary, a model including complete loss of cccDNA during cellular proliferation reproduces HBV viremia reasonably well without resulting in liver destruction.

### 3.5. Model Robustness

#### 3.5.1. Sensitivity to Viral Infectivity

In order to determine the sensitivity of our results to the fixed value of the virus infectivity parameter, k, we varied it in the range [0.1k , 2k], where $k=0.55\times 10^{-10}$ while keeping the value of the other parameters constant. We then determined the impact of this change on outputs such as HT and (%) lowest liver cell number (LCN) for patients P1 to P6 using models *M1*, *M2*, *M3*. The results are summarized in Tables S2–S4.

We found that both HT and LCN were only slightly affected by the change in virus infectivity for all patients except P1 under all models (Tables S2–S4). Models *M2* and *M3*, where an infected cell proliferates to generate at least one infected cell, result in a non-functioning liver for at least one of the 6 patients for all values of virus infectivity (Tables S3 and S4). In fact, *M2* had the most cases of violations of these criteria of liver non-destruction (Table S3) followed by *M3* (Table S4). In contrast, *M1* results in a functioning liver for all patients, although for P1 this is true only for values of virus infectivity $k\leq 0.55\times 10^{-10}$ (Table S2). This is because we kept the values of parameters δ, $p_{V}$, $c_{V}$ and r fixed at their estimates obtained with $k=0.55\times 10^{-10}$.

However, if we refit the data for each value of the virus infectivity parameter in the range [0.1k , k], *M1* (but not *M2* and *M3*) results in a functioning liver for all patients for all values of virus infectivity (shown for P1 in Table S5). All values of $k<0.55\times 10^{-10}$ can also be considered as cases of reduction in viral infectivity from its default value $k=0.55\times 10^{-10}$, possibly due to the emergence of HBV-specific antibodies. Simulations showed that there is less liver injury (HT decreases while LCN increases) as we decrease viral infectivity under all models; however the AIC_C_ increases at the same time. In conclusion, only model *M1* results in a functioning liver for all values of k while clearing acute HBV infection. If the reduction in viral infectivity is less than 50%, then model *M3* also fails to result in a functioning liver while model *M2* always fails even if the reduction in viral infectivity is as high as 90%. Even though model *M3* results in a functioning liver if the reduction in the viral infectivity is more than 50%, it still performs worse than model *M1* with no reduction in the viral infectivity (see AIC_C_ in Table S5).

#### 3.5.2. Sensitivity to the Initial Infected Cell Population

We showed that *M1* is the only model that results in the clearance of the acute HBV infection with a functioning liver and lowest AIC_C_. Therefore, we used *M1* to analyze the model robustness relative to the initial infected cell population at the viremia peak. We varied the peak infected cell population between 85% and 99% of the initial hepatocyte population [66]. We also tested the robustness of the model against an extreme case where the peak infected cell population is 50% of the initial hepatocyte population.

We found that a change in the initial infected cell population has very little impact on HT and LCN for all six patients (Table S9). Similar to the case above, we performed best-fit estimates for the values of the parameters δ, $p_{V}$, $c_{V}$ and r for all different initial infected cell population choices, and we found that in all cases for all patients *M1* resulted in a functioning liver.

### 3.6. Optimal Parameter Values for the 6 Acutely HBV Infected Patients

**Figure 2:**
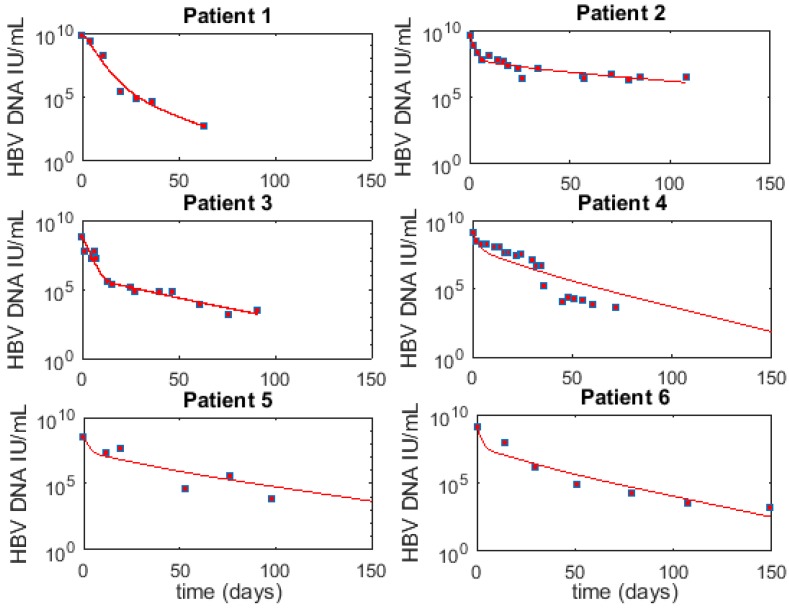
Best fit (line) to HBV viremia (squares) for six patients using *M1*. The corresponding parameter values are given in Table 3.

**Figure 3:**
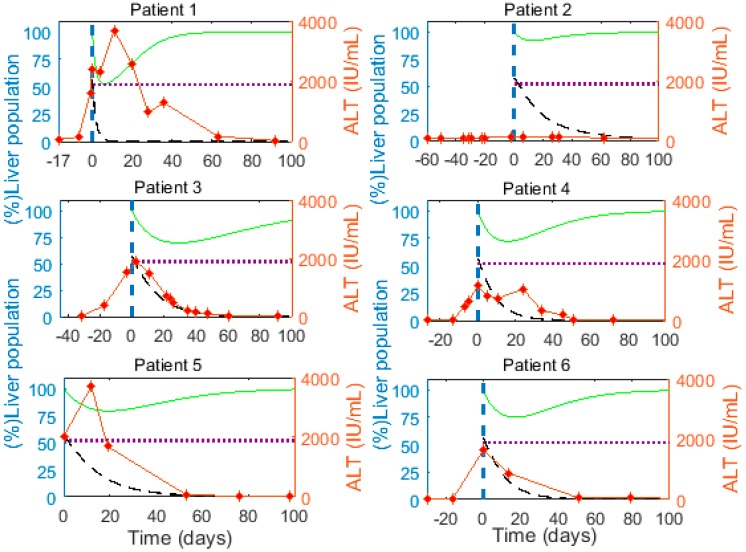
(Left) Y-axis: Predicted (%) infected cell population (I, black dashed line) and (%) total liver cell population (100(I+T+θH)/H, green solid line) calculated from *M1* using the best-fit parameters in Table 3. The dotted horizontal line represents the critical threshold for the liver cell population during the process of infection clearance, which is 52% of H. (Right) Y-axis: Alanine aminotransferase (ALT, IU/mL) measured in patients (red diamond markers and red line). The vertical dashed line represents time 0, when we start analyzing/fitting the viral load.

Using the best fits results for each patient under *M1* and I(0)=0.95×0.6H, we report parameters for individual patients in Table 3 (the associated HBV viremia fit and cell populations are given in Figure 2 and Figure 3, respectively). The median (range) for parameter δ is 0.065/day (0.03–0.58), while $p_{V}$ is 4.55 virions/cell day (0.06–788), $c_{V}$ is 0.75/day (0.67–0.92), r is 0.10/day (0.05–0.24) and HT is 0.825 (0.72–1.26).

We observe a large decline (≥25%) in the total liver cell population close to the peak of HBV viremia in 5 of the 6 patients (Figure 3, green curve). This reduction in the liver cell population implies that infected cells are lost faster than the remaining cells can proliferate to compensate for the lost liver mass. The elevated alanine aminotransferase (ALT) levels as measured in the patients range between 130 and 3709 IU/mL (red markers, Figure 3), and peak near the time the model predicts major hepatocyte loss in almost all patients, supporting the model prediction of significant loss of (infected) cells. The peak ALT levels in all patients except patient 2 were >1000 IU/mL, which is 15 times the upper limit of the normal range (i.e., 19 IU/mL for women and 30 IU/mL in men [82]). ALT levels greater than 15 times the upper limit of the normal range indicate severe acute liver cell injury [83], consistent with the model prediction of substantial loss of hepatocytes near the ALT peaks (Figure 3). Lastly, Guidotti et al. [9] observed that in acute HBV infection of chimpanzees a larger fraction of hepatocytes were ongoing apoptosis than were in mitosis a few weeks after the viral load peak, also consistent with hepatocyte loss.

## 4. Discussion

The immune system plays a very important role in the process of HBV infection clearance [41,42]. The antiviral immune response involves T cells, which kill infected cells and secrete cytokines responsible for the inhibition of HBV replication, as well as B cells that secrete antibodies that neutralize the virus [54]. An inefficient and weak immune response is associated with failure to clear acute HBV infection and results in chronicity of HBV infection [84,85,86]. However, the clearance of acute HBV infection in humans is intriguing as: (i) HBV infects nearly all hepatocytes at the peak of acute infection [4,22,55,56], (ii) infected hepatocytes contain at least one cccDNA molecule, but possibly more due to the transport of relaxed circular DNA (rcDNA) to the nucleus [25,87,88], (iii) template (cccDNA) has a long half-life of around 7–8 weeks [57], and (iv) there is limited HBV-specific CTL precursor frequency which can clear HBV infection [4]. And, yet clearance occurs in 8 to 12 weeks post-peak of infection [54,55]. Summers et al. have estimated that in woodchucks clearance of WHV (the woodchuck hepatitis virus model of HBV) infection could be achieved with liver turnover (including HT and compensatory proliferation) as low as 0.7 [77]. However, these estimates are the lower bound and the actual estimates could be more than 3 times these lower bounds due to the inefficiency of DNA extraction in the experiments [53]. A modeling study also estimated the minimum liver turnover at 2.6 in resolving infection when there is only cytolytic death of infected cells and cccDNA survives the process of cellular proliferation [14]. Furthermore, an agent-based modeling study found that despite high HT, the cytolytic response alone is incapable of resolving acute HBV infection [24]. It should be noted that high HT implies high levels of death of HBV-infected hepatocytes, which has important biological side-effects such as potential clonal expansion of mutant and epigenetically altered hepatocytes [89].

Another key aspect of the immune response against HBV involves non-cytolytic effects (through cytokines) that inhibit intracellular replication. Non-cytolytic mechanisms when added to the cytolytic response significantly reduce HT and the burden on cytolytic cells by lowering the number of infected cells required to be killed by each *T* cell each day [24,25,53]. However, we found that even cytolytic and non-cytolytic mechanisms acting together fail to resolve infection in a time-frame of 8 to 12 weeks in recently conducted agent based 2-D and 3-D modeling studies of HBV infection [24,25]. Additional mechanisms, such as the loss of cccDNA during cellular proliferation of infected cells and cytokine-mediated cure of infected cells, may significantly contribute towards the immune-mediated clearance of acute HBV infection [9,44,48,49].

Here we investigated systematically both the effect of proliferation of infected cells and cytokine-mediated cure of infected cells. A number of in vitro and in vivo experiments have hinted that cccDNA and other intracellular HBV intermediates are lost during cellular proliferation [50,51,52]. One mechanism proposed to explain this phenomenon was that there is not enough time for rcDNA to convert to cccDNA during rapid cell proliferation [51,90]. Another possible explanation is that cccDNA is released from the nucleus when the nuclear membrane is disrupted during mitosis and fails to be reincorporated in the nucleus when the nuclear membrane reforms [53,61]. An in vivo study by Ozer et al. [91] also showed an inverse correlation between the expression of proliferating cell nuclear antigen (PCNA) and the presence of episomal HBV DNA in individual hepatocytes. Recently, Dandri and Petersen also suggested that hepatocyte proliferation may accelerate cccDNA decline [92]. Moreover, an in vivo study in humanized mice, which employed an advanced (and precise) technique for measuring cccDNA, concluded that cccDNA is lost during cellular proliferation [50]. Some modeling studies also analyzed the importance of proliferation-induced loss of cccDNA during clearance of acute HBV infection in chimpanzees [26] and in ducks [24,25].

Our data fitting suggested that cellular proliferation of infected cells is more likely to produce two uninfected cells than one or two infected progeny. When we assumed that cccDNA is preserved or only partially lost during cellular proliferation, clearance became more dependent on cell death and the liver size often reduced to levels compromising its integrity. This was more prominently evident in patient 1 where HBV viremia declined rapidly and became undetectable within 60 days of the viremia peak. The mean proliferation rate among all patients was found to be 0.13/day, lower than previous estimates [5] but much higher than the proliferation rate of quiescent hepatocytes [70]. The requirement for rapid proliferation during clearance is obvious, as it is desirable to make up for the loss of liver mass, which results from death of infected cells. This rapid proliferation is enabled by cytokines such as IFN-γ secreted by CD^4+^ T cells (in particular T-helper 1 cells), which induces TNF-α and interleukin-6 (IL-6) that promote hepatocyte growth factor [84]. These cytokines have been observed in adult individuals after acute HBV infection but not in chronic HBV patients [84].

Our simulations showed a significant decrease of up to nearly 80% in the hepatocyte population for one patient under *M1* during infection clearance. Even with such a significant decrease in hepatocytes, the total liver cell number always remains greater than 52% of the number of cells in a normal liver. Significant loss of hepatocytes is expected during acute HBV infection as ALT levels reach very high levels (>15 times the normal upper limit), indicating severe acute liver cell injury [83]. Additionally, Guidotti et al. [9] observed that in acute HBV infection of chimpanzees a larger fraction of hepatocytes were ongoing apoptosis than were in mitosis a few weeks after the viral load peak, also consistent with hepatocyte loss. The large decrease in the hepatocyte number in the simulations could also be expected since the clearance of acute woodchuck hepatitis virus infection could involve hepatocyte loss of up to 3 liver equivalents in a short period of time [14,24,25,53,77]. However, we do not know of any experimental studies that explicitly confirm this large loss, in animal models or humans. Future studies are needed to provide more quantitative information about the degree of liver loss during acute HBV infection.

Our estimate of the mean half-life of infected cells of 2.1 days during the clearance of acute HBV infection was similar to the estimate made by Whalley et al. of 3.7 ± 1.5 days [6]. Both estimates are much shorter than previous estimates ranging between 10–100 days during chronic HBV infection under antiviral therapy [93,94]. The longer estimated half-lives of infected hepatocytes during chronic infection could reflect the exhaustion of CD8 effector cells, which makes them less effective killer cells [95,96].

When we analyzed the effect of cytokine-mediated cure of infected cells, we found that it is insufficient to simultaneously fit the measured viral load data and maintain necessary liver mass for all patients, consistent with [24]. We also simulated the case where cccDNA is lost during cellular proliferation together with cytokine-mediated cure of infected cells. However, this case showed no improvement over the model without cytokine-mediated cure of infected cells. In this model, we did not test the possibility that cytokines not only lead to cure of HBV infected cells, but also delay the reinfection of newly cured cells.

The details of the control of liver cell proliferation during infection and clearance are not fully known and it is unclear if they are similar to that seen after liver resection, which is better studied. After liver resection, an increase in liver cell size is the first response to compensate for the liver loss, followed by hepatocyte proliferation resulting in an increase of liver cell number [97,98]. We investigated a model including the effect of an increase in hepatocyte cell size (Supplementary Material Section S1.1). In this model, we first allow an increase in the cell size as a compensatory process for the lost liver mass during the infection clearance and found that the major difference was the reduction in LCN in all patients (Table S10).

Other mechanisms could also be studied. We have previously analyzed the possible impact of HBV-specific antibodies in the control of HBV infection, as well as the effect of other putative immune responses [5,32]. In addition, one could explore the possibility of different proliferative capacities of infected and uninfected hepatocytes. However, the probability of a huge difference in the proliferation rate of infected and uninfected cells is small, as one study found that the recovered liver primarily consists of cells derived from the infected cell population [14]. Models have been developed to test the possibility that cytokines not only lead to cure of HBV infected cells but also delay reinfection of newly cured cells [32,66]. Lastly, combinations of these mechanisms, the most likely scenario in vivo, could be investigated. However, these more complex models will increase the number of parameters and make it more difficult to fit the data adequately and interpret the results. For this reason, additional experimental data related to the clearance of acute HBV infection in humans will further improve such analysis.

In conclusion, clearance of acute HBV infection is likely dependent on cellular proliferation of an infected cell resulting in two uninfected cells.

## Figures and Tables

**Table 1:** Hepatocyte turnover (HT) and (%) Lowest liver cell number (LCN) under models *M1*, *M2* and *M3*. Here, LCN represents the lowest liver cell number during the process of infection clearance as a fraction of H. The critical LCN threshold to maintain liver integrity is 52%. Bold and underlined numbers represents the case of liver-destruction and/or loss of liver integrity.

Model	Patient 1	Patient 2	Patient 3	Patient 4	Patient 5	Patient 6
*M1*						
HT	1.26	0.72	0.83	0.84	0.78	0.82
LCN	52.6	91.8	69.3	72.4	79.3	75
*M2*						
HT	1.5	1.27	1.02	1.0	1.05	1.01
LCN	**43.8**	**51.1**	**44.1**	**42.9**	**45.5**	**43.5**
*M3*						
HT	1.34	0.98	0.99	1.0	0.98	0.99
LCN	**49.6**	83.5	98.3	64.8	66	68.5

**Table 2:** Hepatocyte turnover (HT), (%) Lowest liver cell number (LCN), non-destructive turnover (NDT) under models *M4* and *M5*. Here, LCN represents the lowest liver cell number as a fraction of H during the process of infection clearance and NDT denotes the number of cured hepatocytes measured in original hepatocyte number equivalents. The critical threshold of the liver cell number to maintain liver integrity is 52%. Bold and underlined figures represents the case of liver-destruction and/or non-functionality of the liver.

Model	Patient 1	Patient 2	Patient 3	Patient 4	Patient 5	Patient 6
*M4*						
HT	1.49	0.16	0.12	0.01	0.02	0.03
LCN	**43.8**	93.6	98.2	99.5	98.9	99.8
NDT	0.001	0.851	0.905	1.01	0.963	0.984
*M5*						
HT	1.26	0.69	0.7	0.84	0.62	0.79
LCN	52.7	92.3	98.5	72.1	98.8	76.7
NDT	0.002	0.047	0.008	0.006	0.113	0.029

**Table 3:** Parameters for best fits for six patients using *M1*. The parameters δ, $p_{V}$, $c_{V}$, r, HT and AIC_C_ represent the infected cell loss rate, the per infected cell production rate of HBV, the clearance rate of HBV, the proliferation rate, hepatocyte turnover and corrected Akaike information criterion, respectively.

Patient	δ/Day	$p_{V}$ Vir/Cell·Day	$c_{V}$/Day	r/Day	HT	AIC_C_
Patient 1	0.58	788	0.92	0.20	1.26	14.08
Patient 2	0.03	5.7	0.83	0.24	0.72	−29.31
Patient 3	0.06	0.06	0.67	0.05	0.83	−15.66
Patient 4	0.09	5.30	0.67	0.10	0.84	9.94
Patient 5	0.05	1.80	0.67	0.10	0.78	23.96
Patient 6	0.07	3.8	0.87	0.10	0.82	19.77
Median	0.065	4.55	0.75	0.10	0.825	

## Supplementary Materials

The following are available online at www.mdpi.com/1999-4915/9/11/350/s1.
