# Supplementary material for: The Role of Infected Cell Proliferation in the Clearance of Acute HBV Infection in Humans
Source: Viruses. 2017 Nov 18;9(11):350. doi: 10.3390/v9110350 (PMC5707557; doi:10.3390/v9110350)
Supplement: Supplementary file 1 [file viruses-09-00350-s001.pdf]

Supplementary Information

The role of infected cell proliferation in the clearance of acute HBV infection in humans

Ashish Goyal [1], Ruy M. Ribeiro [1,2] and Alan S. Perelson [1]

[1], Theoretical Biology and Biophysics, Los Alamos National Laboratory, USA

[2], Laboratório de Biomatemática, Faculdade de Medicina da Universidade de Lisboa, Portugal

1. Mathematical Models

1.1 Model that allows accounting for liver cell size increase (*M6*)

In this section, we propose a new model that also account for cell size increase. After liver resection, the first response to compensate for lost liver mass is cell size increase [1, 2]. Cellular proliferation only occurs when the total liver cell population goes below 70% of the initial cell population [1]. To simulate this, we replace $\left(1-\frac{T+I+\theta H}{H}\right)$ by $\max(0,\left(1-\frac{T+I+\theta H}{0.7H}\right))$ in *M1* (in the main text) and refer to it as *M6*. This model assume that an increase in hepatocyte size can compensate for lower cell numbers, without critically affecting the function of the liver [1]. Additionally, to keep the models simple, we assume that an increase in size of the hepatocytes does not have substantial impact on virus infectivity (k), virus productivity (p_V) and the death rate of infected cells (δ). This model was simulated assuming 60% hepatocyte population (*i.e.*, $\theta = 0.4$). The equations for this model are

$$\frac{dT}{dt} = r(T+2I)\max(0,\left(1-\frac{T+I+\theta H}{0.7H}\right)) - kVT$$

$$\frac{dI}{dt} = kVT - \delta I - rI\max(0,\left(1-\frac{T+I+\theta H}{0.7H}\right))$$

$$\frac{dV}{dt} = p_V I - c_V V$$

Results of this model are presented in Table S9. Essentially, we found that the major differences are, (i) the reduction in lowest liver cell number (LCN), and (ii) an increase in HT for all patients except patient 1, when we first allow an increase in the cell size as a compensatory process for the lost liver mass during the infection clearance.

2. Results

Tables:

Table S1: HBV viral data of 6 acutely infected patients. (-) represents that the data was not measured at that time point.

Time (day)	P1 (log IU/mL)	P2 (log IU/mL)	P3 (log IU/mL)	P4 (log IU/mL)	P5 (log IU/mL)	P6 (log IU/mL)
0	9.82	9.58	8.80	9.09	8.51	9.06
1	-	-	7.73	-	-	-
2	-	8.84	-	8.46	-	-
4	9.33	8.28	-	-	-	-
5	-	-	7.19	8.27	-	-
6	-	7.77	7.76	-	-	-

7	-	-	7.26	8.24	-	-
10	-	8.08	-	-	-	-
11	8.20	-	-	8.04	-	-
12	-	-	-	-	7.35	-
13	-	-	5.62	8.06	-	-
14	-	7.76	-	-	-	7.98
15	-	-	5.40	-	-	-
16	-	-	-	7.63	-	-
17	-	7.68	-	-	-	-
18	-	-	-	7.59	-	-
19	-	7.32	-	-	7.68	-
20	5.36	-	-	-	-	-
22	-	-	-	7.41	-	-
24	-	7.16	-	7.52	-	-
25	-	-	5.11	-	-	-
26	-	6.40	-	-	-	-
27	-	-	4.86	-	-	-
28	4.83	-	-	-	-	-
30	-	-	-	7.13	-	6.13
32	-	-	-	6.67	-	-
34	-	7.14	-	6.66	-	-
36	4.65	-	-	5.24	-	-
40	-	-	4.89	-	-	-
45	-	-	-	4.05	-	-
47	-	-	4.82	-	-	-
48	-	-	-	4.42	-	-
51	-	-	-	4.29	-	4.88
53	-	-	-	-	4.57	-
55	-	-	-	4.11	-	-
56	-	6.53	-	-	-	-
57	-	6.36	-	-	-	-
60	-	-	-	3.82	-	-
61	-	-	3.93	-	-	-
63	2.68	-	-	-	-	-
71	-	6.64	-	-	-	-
72	-	-	-	3.65	-	-
76	-	-	3.15	-	5.64	-
79	-	6.28	-	-	-	4.30
85	-	6.44	-	-	-	-
91	-	-	3.50	-	-	-
98	-	-	-	-	3.88	-
107	-	-	-	-	-	3.48
108	-	6.50	-	-	-	-
149	-	-	-	-	-	3.20
152	-	-	-	4.08	-	-
169	-	-	-	-	3.96	-

Table S2: Sensitivity to the virus infectivity (k): Hepatocyte turnover (HT) and (%) lowest liver cell number (LCN) under the model *M1*. The critical threshold of the liver cell population to maintain integrity is 52%. Bold and underlined figures represents the case of liver-destruction and/or non-functionality of the liver. Here, $k_1 = 0.55 \times 10^{-10}$ mL/copies·day.

Patient	$k = 0.1k_1$	$k = 0.5k_1$	$k = 1k_1$	$k = 1.5k_1$	$k = 2.0k_1$
**P1**					
HT	1.19	1.25	1.26	1.61	2.43
LCN	55.9	53.3	52.6	**48.8**	**47.4**
**P2**					
HT	0.70	0.71	0.72	0.76	0.79
LCN	92	91.9	91.8	91.7	91.7
**P3**					
HT	0.83	0.83	0.83	0.84	0.84
LCN	69.5	69.5	69.3	69.3	69.2
**P4**					
HT	0.83	0.84	0.84	0.86	0.86
LCN	72.5	72.3	72.4	71.9	71.7
**P5**					
HT	0.78	0.78	0.78	0.79	0.79
LCN	79.3	79.2	79.3	79.1	79
**P6**					
HT	0.81	0.81	0.82	0.83	0.83
LCN	75.1	75	75	74.7	74.6

Table S3: Sensitivity to the virus infectivity (k): Hepatocyte turnover (HT) and (%) lowest liver cell number (LCN) under the model *M2*. The critical threshold of the liver cell population to maintain integrity is 52%. Bold and underlined figures represents the case of liver-destruction and/or non-functionality of the liver. Here, $k_1 = 0.55 \times 10^{-10}$ mL/copies·day.

Patient	$k = 0.1k_1$	$k = 0.5k_1$	$k = 1k_1$	$k = 1.5k_1$	$k = 2.0k_1$
**P1**					
HT	1.45	1.49	1.5	1.51	1.51
LCN	**47.3**	**44.4**	**43.8**	**41.1**	**40.5**
**P2**					
HT	1.25	1.27	1.27	1.31	0.32
LCN	52.8	**51.5**	**51.1**	**48.8**	**47.7**
**P3**					
HT	1.01	1.02	1.02	1.02	1.02
LCN	**44.2**	**44.1**	**44.1**	**43.8**	**43.7**
**P4**					
HT	1	1	1	1.01	1.02
LCN	**43.2**	**43**	**42.9**	**42.5**	**42.2**
**P5**					
HT	1.05	1.05	1.05	1.05	1.05
LCN	**45.6**	**45.5**	**45.5**	**45.2**	**45**
**P6**	1	1	1.01	1.01	1.02
HT	**43.7**	**43.5**	**43.5**	**43.1**	**42.9**

LCN					

Table S4: Sensitivity to the virus infectivity (k): Hepatocyte turnover (HT) and (%) lowest liver cell number (LCN) under the model *M3*. The critical threshold of the liver cell population to maintain integrity is 52%. Bold and underlined figures represents the case of liver-destruction and/or non-functionality of the liver. Here, $k_1 = 0.55 \times 10^{-10}$ mL/copies·day.

Patient	$k = 0.1k_1$	$k = 0.5k_1$	$k = 1k_1$	$k = 1.5k_1$	$k = 2.0k_1$
**P1**					
HT	1.28	1.33	1.34	1.57	2.52
LCN	**<u>51.9</u>**	**<u>50.2</u>**	**<u>47.8</u>**	**<u>46.2</u>**	**<u>45.1</u>**
**P2**					
HT	0.96	0.98	0.98	1.03	1.05
LCN	83.7	83.5	83.5	83.1	83
**P3**					
HT	0.98	0.99	0.99	1	1
LCN	98.3	98.3	98.3	98.3	98.3
**P4**					
HT	0.99	1	1	1.01	1.02
LCN	65	64.8	64.8	64.3	64
**P5**					
HT	0.98	0.98	0.98	0.98	0.99
LCN	65.9	65.9	66	65.7	65.7
**P6**					
HT	0.99	0.99	0.99	1	1
LCN	68.6	68.5	68.5	68.3	68.2

Table S5: Parameters for best fits for patient 1 using *M1*, *M2* and *M3* for different values of virus infectivity (k). The parameters δ, p_V, c_V, r, HT, LCN and AICc represent the infected cell loss rate, the per infected cell production rate of HBV DNA particles, the clearance rate of HBV particles, the proliferation rate, hepatocyte turnover, (%) lowest liver cell number and corrected Akaike information criterion, respectively. The critical threshold of the hepatocyte population to maintain liver integrity is 52%. Bold and underlined figures represents the case of liver-destruction and/or non-functionality of the liver. Here, $k_1 = 0.55 \times 10^{-10}$ mL/copies·day. Numbers in color red represent the case of lowest AICc and it happened to be $k = 0.55 \times 10^{-10}$ mL/copies·day for all models, which we chose as the default value in the main text.

Model	$k = 0.1k_1$	$k = 0.3k_1$	$k = 0.5k_1$	$k = 0.7k_1$	$k = 0.9k_1$	$k = 1k_1$
M1						
HT	0.88	0.89	1.13	1.18	1.23	1.26
LCN	73.98	73.51	58.1	55.88	53.65	52.6
AICc	20.32	20.30	19.13	16.42	14.57	14.08
M2						
HT	1.09	1.1	1.11	1.45	1.48	1.5
LCN	**<u>44.59</u>**	**<u>44.21</u>**	**<u>43.9</u>**	**<u>44.51</u>**	**<u>43.97</u>**	**<u>43.8</u>**
AICc	21.06	21.06	21.06	20.24	17.16	15.69
M3						

HT	1.05	1.07	1.24	1.28	1.31	1.34
LCN	95.97	95.98	**52.91**	**51.52**	**50.35**	**47.8**
AICc	21.07	21.07	21.02	17.98	15.37	14.43

Table S6: Corrected Akaike information criterion (AICc) comparison for models - *M1*, *M2* and *M3*.

**Patient**	***M1***	***M2***	***M3***
Patient 1	14.08	15.69	14.43
Patient 2	-29.31	-27.77	-26.98
Patient 3	-15.66	-15.61	-15.62
Patient 4	9.94	13.1	12.94
Patient 5	23.96	24.99	25.02
Patient 6	19.77	21.48	21.46
**Total AICc**	45.76	50.51	50.26

Table S7: Parameters for best fits for patient 1 using *M1*, *M2* and *M3*. The parameters δ, p_V, c_V, r, HT and AICc represent the infected cell loss rate, the per infected cell production rate of HBV DNA particles, the clearance rate of HBV particles, the proliferation rate, hepatocyte turnover and corrected Akaike information criterion respectively.

Model	δ /day	p_V vir/cell.day	c_V /day	r /day	HT	AICc
M1	0.578	787.81	0.92	0.195	1.26	14.08
M2	0.668	626.07	0.73	0.29	1.5	15.69
M3	0.596	818.16	0.95	0.215	1.34	14.43

Table S8: Parameters for best fits for six patients using *M4*. The parameters δ, p_V, c_V, r and ρ represent, the infected cell loss rate, the per infected cell production rate of HBV DNA particles, the clearance rate of HBV particles, the proliferation rate and the rate of cytokine-mediated cure of infected cells, respectively.

Patient	δ /day	p_V vir/cell.day	c_V /day	r /day	ρ /day
Patient 1	0.667	627.64	0.73	0.289	0.0005
Patient 2	0.006	4.06	0.78	0.014	0.0305
Patient 3	0.008	0.05	0.67	0.205	0.0606
Patient 4	0.001	2.96	0.68	0.028	0.0902
Patient 5	0.001	1.66	0.86	0.011	0.054
Patient 6	0.002	2.22	0.91	0.8	0.0736

Table S9: Sensitivity to the value of the initial infected cell population ($I(0)$): Hepatocyte turnover (HT) and (%) Lowest liver cell number (LCN) under the model *M1*. The critical threshold of the liver population to maintain liver integrity is 52%. Bold and underlined figures represents the case of liver-destruction and/or non-functionality of the liver. Here, $I(0) = (1-\theta)\phi H$, where $\theta = 0.4$.

Patient	$\phi = 0.5$	$\phi = 0.85$	$\phi = 0.87$	$\phi = 0.89$	$\phi = 0.91$	$\phi = 0.93$	$\phi = 0.95$	$\phi = 0.97$	$\phi = 0.99$
**P1**									

HT<br>LCN	0.96<br>65.6	1.2<br>55.5	1.21<br>54.9	1.23<br>54.4	1.24<br>53.8	1.25<br>53.2	**1.26**<br>**52.6**	1.28<br>52.1	1.29<br>**<u>51.5</u>**
**P2**<br>HT<br>LCN	 0.51<br>94.8	 0.68<br>92.5	 0.69<br>92.4	 0.7<br>92.3	 0.7<br>92.1	 0.71<br>92	 **0.72**<br>**91.8**	 0.73<br>91.8	 0.74<br>91.6
**P3**<br>HT<br>LCN	 0.49<br>84.1	 0.76<br>72.9	 0.78<br>72.2	 0.79<br>71.5	 0.81<br>70.8	 0.82<br>70.1	 **0.83**<br>**69.3**	 0.85<br>68.7	 0.86<br>68
**P4**<br>HT<br>LCN	 0.52<br>84.8	 0.78<br>75.2	 0.79<br>74.6	 0.8<br>74.1	 0.82<br>73.5	 0.83<br>72.9	 **0.84**<br>**72.4**	 0.85<br>71.7	 0.87<br>71.1
**P5**<br>HT<br>LCN	 0.47<br>89.1	 0.72<br>81.5	 0.73<br>81	 0.74<br>80.6	 0.75<br>80.1	 0.77<br>79.7	 **0.78**<br>**79.3**	 0.79<br>78.7	 0.8<br>78.3
**P6**<br>HT<br>LCN	 0.5<br>86.5	 0.75<br>77.6	 0.76<br>77.1	 0.78<br>76.6	 0.79<br>76	 0.80<br>75.5	 **0.82**<br>**75**	 0.83<br>74.4	 0.84<br>73.9

Table S10: Hepatocyte turnover (HT) and (%) lowest liver cell number (LCN). Here, LCN represents the lowest hepatocyte cell number divided by *H* during the process of infection clearance under the model *M6*. Accounting for an increase in hepatocyte size by 1.4 times, the critical value of LCN is now 48.6%. Bold and underlined figures represents the case of liver-destruction and/or non-functionality of the liver.

MODEL	PATIENT 1	PATIENT 2	PATIENT 3	PATIENT 4	PATIENT 5	PATIENT 6
M6<br>HT<br>LCN	 1.17<br>49.7	 0.83<br>69.4	 0.90<br>56.9	 0.91<br>59.5	 0.87<br>63.6	 0.86<br>68.8

<u>References:</u>

1. Miyaoka, Y.; Miyajima, A., To divide or not to divide: revisiting liver regeneration. *Cell Division* **2013,** 8, (1), 8.
2. Nagasue, N.; Yukaya, H.; Ogawa, Y.; Kohno, H.; Nakamura, T., Human liver regeneration after major hepatic resection. A study of normal liver and livers with chronic hepatitis and cirrhosis. *Annals of Surgery* **1987,** 206, (1), 30-39.
